# Greater cognitive reserve is related to lower cortical excitability in healthy cognitive aging, but not in early clinical Alzheimer’s disease

**DOI:** 10.3389/fnhum.2023.1193407

**Published:** 2023-07-27

**Authors:** Stephanie S. Buss, Peter J. Fried, Joanna Macone, Victor Zeng, Emma Zingg, Emiliano Santarnecchi, Alvaro Pascual-Leone, David Bartrés-Faz

**Affiliations:** ^1^Division of Cognitive Neurology, Berenson-Allen Center for Noninvasive Brain Stimulation, Beth Israel Deaconess Medical Center, Boston, MA, United States; ^2^Department of Neurology, Harvard Medical School, Boston, MA, United States; ^3^Department of Psychiatry, Beth Israel Deaconess Medical Center, Boston, MA, United States; ^4^Program of All-inclusive Care for the Elderly (PACE), Cambridge Health Alliance, Cambridge, MA, United States; ^5^Precision Neuromodulation Program, Gordon Center for Medical Imaging, Department of Radiology, Massachusetts General Hospital, Harvard Medical School, Boston, MA, United States; ^6^Deanna and Sidney Wolk Center for Memory Health, Hinda and Arthur Marcus Institute for Aging Research, Hebrew SeniorLife, Boston, MA, United States; ^7^Department of Medicine, Faculty of Medicine and Health Sciences, Institute of Neurosciences, University of Barcelona, Barcelona, Spain; ^8^Institut d’Investigacions Biomèdiques August Pi i Sunyer (IDIBAPS), Barcelona, Spain

**Keywords:** cognitive reserve, transcranial magnetic stimulation, excitability, aging, Alzheimer’s disease

## Abstract

**Objective:**

To investigate the relationship between cortico-motor excitability and cognitive reserve (CR) in cognitively unimpaired older adults (CU) and in older adults with mild cognitive impairment or mild dementia due to Alzheimer’s disease (AD).

**Methods:**

Data were collected and analyzed from 15 CU and 24 amyloid-positive AD participants aged 50–90 years. A cognitive reserve questionnaire score (CRQ) assessed education, occupation, leisure activities, physical activities, and social engagement. Cortical excitability was quantified as the average amplitude of motor evoked potentials (MEP amplitude) elicited with single-pulse transcranial magnetic stimulation delivered to primary motor cortex. A linear model compared MEP amplitudes between groups. A linear model tested for an effect of CRQ on MEP amplitude across all participants. Finally, separate linear models tested for an effect of CRQ on MEP amplitude within each group. Exploratory analyses tested for effect modification of demographics, cognitive scores, atrophy measures, and CSF measures within each group using nested regression analysis.

**Results:**

There was no between-group difference in MEP amplitude after accounting for covariates. The primary model showed a significant interaction term of group*CRQ (R^2^_adj_ = 0.18, *p* = 0.013), but no main effect of CRQ. Within the CU group, higher CRQ was significantly associated with lower MEP amplitude (R^2^_adj_ = 0.45, *p* = 0.004). There was no association in the AD group.

**Conclusion:**

Lower cortico-motor excitability is related to greater CRQ in CU, but not in AD. Lower MEP amplitudes may reflect greater neural efficiency in cognitively unimpaired older adults. The lack of association seen in AD participants may reflect disruption of the protective effects of CR. Future work is needed to better understand the neurophysiologic mechanisms leading to the protective effects of CR in older adults with and without neurodegenerative disorders.

## 1. Introduction

Cognitive reserve (CR) is a construct proposed to explain why the severity of brain pathology in many neurological disorders does not always predict cognitive impairment (Stern, 2009). CR is accumulated over a lifetime through a variety of educational, occupational, physical, social, and leisure experiences. In Alzheimer’s disease (AD), high CR may delay the onset of cognitive decline compared to what is expected in patients with elevated brain amyloid and tau accumulation (Soldan et al., 2017). While there is currently no direct measure of CR, a commonly accepted operational definition includes: (1) an assessment of a brain insult impacting cognition, (2) an assessment of cognitive function, and (3) measurement of a variable which influences (1) and (2) [adapted from Stern et al., 2022]. Within this context, imaging techniques including functional MRI (fMRI), spectroscopy, and EEG are well suited to investigate the “neural implementation of” CR in humans, for example elucidating interindividual differences in *efficiency* of brain networks’ activation during cognitive tasks, or *compensatory* processes in response to brain aging or pathology (Stern et al., 2020).

Transcranial magnetic stimulation (TMS) is a neurophysiologic tool that can be used to assess the brain’s response to induced electric current. Applying TMS pulses to primary motor cortex (M1) activates pyramidal neurons (directly or indirectly through inter-neurons) within the corticospinal tract, leading to movements of the contralateral musculature that can be measured as motor evoked potentials (MEPs) using electromyography (EMG). TMS can therefore be used to measure the brain’s response to controlled perturbation, thereby measuring cortico-motor excitability of the stimulated brain region (Kobayashi and Pascual-Leone, 2003; Papeo et al., 2013; Tononi et al., 2016; Ozdemir et al., 2020, 2021), which for example is known to be abnormally increased in AD (Palop and Mucke, 2010; Mimura et al., 2021). Within this context, the Collaborative Framework on Reserve and Resilience reported above emphasized that non-invasive brain stimulation approaches (such as TMS or direct current stimulation) have a relevant role in the study of CR, as they have the ability to probe brain activity associated with CR, potentially leading to insights into the neural- or circuit-based mechanisms subserving CR (Stern et al., 2022).

Based on the above, the present study investigates the relationship between an estimate of CR and TMS M1 measures of cortical excitability in cognitively unimpaired older adults (CU) and in early symptomatic amyloid-positive AD participants (AD). Since increasing cortical excitability may be a parameter related to pathological aging, we hypothesized that higher CR would be related to lower cortical excitability in both groups.

## 2. Materials and methods

### 2.1. Participants

This retrospective study includes 15 CU and 24 AD participants aged 50–90 years old who were enrolled in various clinical research studies at the Berenson-Allen Center for Non-invasive Brain Stimulation, Beth Israel Deaconess Medical Center (BIDMC) from 2011 to 2020. For all studies, participants or their legally authorized representatives signed written informed consent prior to all research procedures in compliance with the Declaration of Helsinki. For the AD group, a diagnosis of mild cognitive impairment (MCI) or mild dementia due to AD was established by an experienced cognitive neurologist in the Cognitive Neurology Unit (CNU) at BIDMC based on history, examination, and cognitive testing. Prior work has shown that clinical diagnosis alone is more than 80% accurate in the diagnosis of AD (Planche et al., 2023). For participants in the clinical research study, the clinical diagnosis of AD was required to be confirmed by biomarker determination. All AD participants included in the present analysis had a documented positive biomarker on Amyloid PET or on cerebrospinal fluid (CSF) testing (Amyloid:Tau Index; ATI). Data was also available for 2 additional AD participants, but since these participants had a “Borderline” ATI or had previously undergone amyloid removal therapy, they were excluded from the present analysis to minimize confounding. Since tau status was not available for participants with only Amyloid PET scans, participants were considered to be A + , with unconfirmed T status, as defined by Jack et al. (2018).

Following the recommendations of the Reserve and Resilience framework, we included the three main components for a CR study: (1) as a measure of brain disease and pathology, AD participants were found to be on the Alzheimer’s continuum with a clinical diagnosis of AD and supportive biomarker evidence; and cortical thickness on MRI was assessed as a measure of atrophy; (2) TMS-based measures cortical excitability were assessed to reflect brain functionality; finally (3) participants were included if a cognitive reserve questionnaire score (CRQ) was recorded as part of the original study, or if participants consented to complete the CRQ retroactively (2018–2019). The CRQ (see below) assesses a broad range of components of CR including education, occupation, leisure activities, physical activity, and social engagement.

All AD and CU participants were excluded if they had a history of unstable medical conditions, a history of neuropsychiatric illness, or contraindication to MRI or TMS. CU participants were also excluded if they had a known history of diabetes, since type-2 diabetes can accelerate cognitive aging and increase the risk of dementia (Biessels et al., 2006).

### 2.2. Cognitive reserve questionnaire

The CRQ was originally developed in Spanish, and has been used in previous CR research to investigate neural mechanisms of CR in both healthy subjects and AD patients (Solé-Padullés et al., 2009; Bosch et al., 2010). The questionnaire was subsequently translated into English and adjusted for cultural differences. For the English version the maximum score is 34 points, with half of the possible points related to childhood education, parental education, and early cultural exposure (for example, exposure to literature, art, and music). Additional questions cover occupation, leisure activities, physical activity, and social engagement. See Supplementary Annex 1.

### 2.3. Neuropsychological assessment

Cognitive testing batteries were administered based on study protocol. Tests were administered by psychometrists trained and supervised by Neuropsychologists in the Cognitive Neurology Unit at BIDMC. To include covariates of global cognition, mood, IQ, and activities of daily living, the following measures were drawn from each study: Mini-Mental Status Examination (MMSE), Geriatric Depression Scale (GDS, 15-item); Weschler Test of Adult Reading (WTAR) (Bright and van der Linde, 2020); and Alzheimer’s Disease Cooperative Study – Activities of Daily Living scale (ADCS-ADL). WTAR was missing for 3 AD participants. GDS was missing from 1 AD participant. ADCS-ADL was missing from 4 AD participants and 5 CU participants.

### 2.4. Amyloid biomarker determination

All AD participants were categorized as Aβ + using either CSF or PET amyloid biomarkers (Palmqvist et al., 2015). Twenty-one AD participants had biomarker determination based on [^18^F]Florbetapir PET (Doraiswamy et al., 2012). Amyloid PET scans were obtained on BIDMC’s Siemens Biograph 64mct multidetector helical PET-CT scanner (Siemens Healthcare). For 20 AD participants, qualitative read for the presence of cortical brain amyloid was performed by a board-certified nuclear medicine specialist to determine Aβ status. For the one AD participant who had previously received aducanumab, amyloid PET positivity was determined by the central reader for the ENGAGE study and confirmed by the site PI.

Five participants had CSF determination based on a clinical CSF cut-off level of Aβ_42_ < 600 (Niemantsverdriet et al., 2017). Lab results were processed via the clinical lab at Athena Diagnostics ADmark Phospho-Tau/Total Tau/A Beta 42 CSF panel. Levels of Aβ_42_ (pg/mL), Phospho Tau (p-Tau, pg/mL), and Total Tau (t-Tau, pg/mL) were recorded for data analysis.

### 2.5. Transcranial magnetic stimulation

All TMS procedures conformed to consensus guidelines for the safe application of TMS endorsed by the International Federation of Clinical Neurophysiology (IFCN) (Rossi et al., 2021). Neuronavigated single-pulse TMS (spTMS) was administered using a figure-of-eight coil (either Nexstim Plc, Finland or MagVenture A/S, Denmark), inducing a monophasic (posterior-anterior) current in the brain. The motor cortex stimulation site (M1) was determined as the location of maximal activation of the right first dorsal interosseous (FDI) in response to stimulation. For all participants, monophasic resting motor threshold (RMT) was measured as the minimum stimulation intensity required to evoke a MEP on at least five out of ten single-pulse TMS trials.

Participants were enrolled in one of three studies from 2011 to 2020 in the Berenson-Allen Center. For all protocols, we delivered monophasic spTMS to M1 at 120% of RMT. MEP peak-to-peak amplitudes (MEP amplitude, μV) were recorded from the FDI using surface EMG and averaged across blocks to assess cortico-motor excitability. To minimize the influence of occasional extreme values, the geometric mean was calculated by taking the arithmetic mean of the log_10_ of individual MEPs. In one study design (Study Design 1; CU *n* = 5; AD *n* = 8), monophasic stimulation was delivered using a MagVenture coil and spTMS trials consisted of the unconditioned pulses from a three-block inter-mixed paired-pulse protocol (total spTMS trials = 45). The second study design (Study Design 2; CU *n* = 10; AD *n* = 18) used a Nexstim coil to deliver single-pulse TMS in three blocks of 35 pulses (total spTMS trails = 105). We controlled for differences in study design in subsequent analyses.

### 2.6. Magnetic resonance imaging

Structural magnetic resonance imaging (MRI) was performed in all participants for use during TMS neuronavigation. Participants received T1-weighted anatomical magnetic resonance imaging scan on a 3T scanner (GE Healthcare, Ltd., UK) using a 3D spoiled gradient echo sequence (Buss et al., 2020). T1-weighted anatomical MRIs were analyzed with Freesurfer 6.0 or 7.1 (documented and freely)^1^ to obtain cortical gray matter thickness (GMT) measurements. One AD participant’s scan was excluded due to a Freesurfer segmentation error.

GMT measurements in all participants (*n* = 15 CU and *n* = 25 AD) was extracted for the left hemisphere mean cortical thickness (average left hemisphere GMT; LH Cortical Thickness) and left precentral thickness (from Desikan-Killiany atlas; Left Precentral Thickness) to serve as covariates in the subsequent nested regression analysis. In order to control for any differences in cortical atrophy, scalp-to-cortex distance (SCD) was measured using Brainsight™ (Rogue Research Inc., Canada) to calculate the in-plane distance between the motor cortex stimulation target and the participant’s scalp along the purported TMS trajectory on each individual’s T1-weighted MRI scan (Brem et al., 2020).

### 2.7. Data management and statistics

Study data were collected and managed using REDCap electronic data capture tools hosted at BIDMC (Harris et al., 2009, 2019). REDCap (Research Electronic Data Capture) is a secure, web-based software platform designed to support data capture for research studies, providing (1) an intuitive interface for validated data capture; (2) audit trails for tracking data manipulation and export procedures; (3) automated export procedures for seamless data downloads to common statistical packages; and (4) procedures for data integration and interoperability with external sources.

JMP Pro 17.0 (SAS Institute Inc., Cary, NC, USA) and Stata 14.2 (StataCorp. 2015) were used for statistical analysis. Power calculations were performed using G*Power 3.1.

#### 2.7.1. Demographics and clinical characteristics

Participant characteristics are reported in the Table 1 the AD and CU groups. For each continuous variable, mean and SD were calculated. For categorical variables, % of total was calculated. Between-group differences were tested using *t*-tests assuming unequal variances for continuous variables and 2-tail Fisher’s Exact test for categorical variables. To control for potential confounders of between-group differences in MEP amplitude, a linear model then assessed the effect of *group* on MEP amplitude accounting for *sex*, *age*, *SCD*, and *study design*.

**TABLE 1:** Participant characteristics.

	CU		AD		df	t ratio	*p*-value
	Value	*n*	Value	*n*			
Age (mean ± SD)*	65.2 ± 9.1	15	70.0 ± 8.4	24	28	−1.7	0.109
Sex (%female)^†^	53.3%	15	41.7%	24	n/a	n/a	0.525
Years of education (mean ± SD)*	16.1 ± 2.3	15	16.9 ± 2.6	23	31	−1.0	0.305
WTAR (age-adjusted IQ)*	118.5 ± 10.7	15	117.4 ± 9.2	21	27	0.3	0.764
MMSE (mean ± SD)*	29.6 ± 0.6	15	25.0 ± 2.8	24	27	7.8	**<0.001**
CRQ total (mean ± SD)*	22.1 ± 4.8	15	22.2 ± 4.4	24	28	−0.2	0.983
MEP amplitude*	587.5 ± 467.1	15	1071.6 ± 895.8	24	36	−2.2	**0.034**
RMT MagPro monophasic*	70.0 ± 10.1	9	63.5 ± 12.4	18	19	1.5	0.162
RMT Nexstim monophasic*	60.6 ± 12.1	15	56.9 ± 9.7	13	26	0.9	0.380
LH cortical thickness (mm)*	2.3 ± 0.1	15	2.2 ± 0.1	23	35	3.0	**0.005**
Left precentral thickness (mm)*	2.5 ± 0.1	15	2.4 ± 0.2	23	34	2.3	**0.027**
Scalp-to-Cortex Distance (mm)*	14.0 ± 3.0	15	14.7 ± 2.1	23	23	−0.7	0.477

Continuous variables are shown as mean ± SD, categorical variables are shown as% of total. Between-group differences were reported using *t-tests assuming unequal variances for continuous variables and ^†^2-tail Fisher’s Exact test for categorical variables. CRQ Total score is out of 34. Although MEP amplitudes were greater in the AD group compared to the CU group a *t*-test, a follow up linear model showed there was no main effect of group on MEP Amplitude when controlling for scalp-to-cortex distance, age, sex, and study design (R^2^_adj_ = 0.08, *p* = 0.160). df, degrees of freedom; MMSE, mini-mental state examination; CRQ, cognitive reserve questionnaire; MEP, motor evoked potential; RMT, resting motor threshold; LH, left hemisphere. Bold values indicate *p*-values < 0.05.

#### 2.7.2. Relationship between CRQ and excitability and power considerations

Our primary analysis used a fixed-effects linear model to test for an effect of *CRQ* on MEP amplitude and included covariates of *sex, age*, *SCD, study design*, *group*, and CRQ*group. However, post-test diagnostics revealed that the primary model residuals were not normally distributed, and thus did not meet the assumptions required for linear modeling. Therefore, a log10 transform was performed on MEP amplitudes (MEP log10). The model was repeated to test for an effect of *CRQ* on MEP log 10 and included covariates of *sex, age*, *SCD, study design*, *group*, and CRQ*group. Type-I error rate (α) was set at 0.05 for this primary model.

For a multiple linear regression model with 7 predictors, our sample of 15 HC and 24 AD gives 80% power to detect a R^2^ ≥ 0.31 corresponding to a large effect.

Since a significant CRQ*group effects was observed (see Results), *post hoc* models were performed in AD and CU separately to test the strength and direction of the association between cognitive reserve and cortical excitability within each group. Separate linear models tested for an effect of *CRQ* on MEP amplitude controlling for *age*. In order preserve statistical power of the *post hoc* models (Austin and Steyerberg, 2015), *age* was included as a covariate because it showed the largest effect in the primary analysis, but other covariates found to be highly insignificant (covariate *p*-values > = 0.475) were not included. Post-test diagnostics showed that the model residuals were not normally distributed for the AD group, so the model was repeated using MEP log 10. Since two separate models were run in this secondary analysis, correction for multiple comparisons was performed using Bonferroni correction, with α corrected to 0.025 for the *post hoc* models (Haynes, 2013).

For a multiple linear regression model with 2 predictors, our sample of 15 HC gives us 80% power to detect an R^2^ ≥ 0.45, while our sample of 24 AD gives us 80% power to detect a R^2^ ≥ 0.32.

#### 2.7.3. Effect of covariates: exploratory analysis

To investigate the effects of demographic variables, cognitive test scores, MRI measurements, and CSF values on the primary model, we performed an exploratory nested regression analysis within each group separately to test how adding each covariates to the model changed the strength of the association between CRQ and MEP amplitude (effect modification).

## 3. Results

### 3.1. Demographics and clinical characteristics

Table 1 shows the demographics, Baseline MEP amplitudes, and CRQ results for the CU and AD participants. There was no difference in CRQ between groups (*p* = 0.983). There were no between-group differences in age, sex, years of education, WTAR, RMT (*p*-values > 0.109). MMSE was lower in the AD group compared to the CU group (*p* < 0.001). The AD group had lower LH cortical thickness (*p* = 0.005) and lower left precentral thickness (*p* = 0.027) compared to the CU group, but there were no between-group differences in SCD (*p* = 0.477). Mean MEP amplitudes were greater in the AD group compared to the CU group (*p* = 0.034, Table 1) using a basic *t*-test. However, there was no main effect of group on MEP Amplitude when controlling for SCD, age, sex, and study design (R^2^_*adj*_ = 0.16, *p* = 0.177).

### 3.2. Relationship between CRQ and excitability

The primary linear model showed a significant interaction term CRQ*group (R^2^_*adj*_ = 0.21, *p* = 0.004) but no main effect of CRQ (*p* = 0.716), showing that the relationship between CR and cortical excitability differed significantly between the AD and CU groups. However, since post-test residuals were not normally distributed (Shapiro-Wilk test, *p* = 0.035), the primary model was re-run with MEP log10 as the dependent variable. In this final model, there remained a significant interaction term CRQ*group (R^2^_adj_ = 0.18, *p* = 0.013) but no main effect of CRQ (*p* = 0.384). Post-test residuals were found to be normally distributed in the model using MEP log10.

**FIGURE 1:**
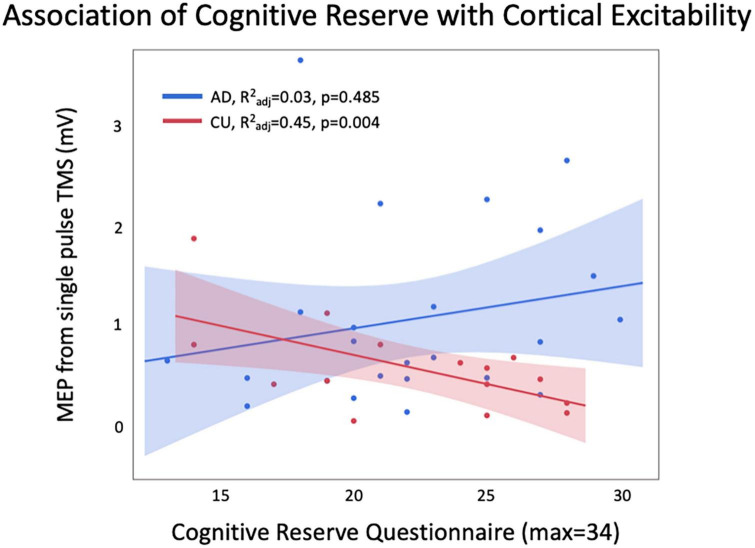
There was a significant relationship between CRQ and MEP amplitude in CU, with higher CRQ related to lower excitability. This relationship was not seen in AD, and a trend was observed in the opposite direction.

Within the CU group, there was a significant relationship between CRQ and cortical excitability (R^2^_adj_ = 0.45, *p* = 0.004; Figure 1) which was not present in the AD group (R^2^_adj_ = 0.03, *p* = 0.485). The significant relationship in the CU group remained significant with Bonferroni correction for 2 comparisons. Post-test diagnostics showed that the model residuals were normally distributed for the CU group, but not for the AD group (Shapiro-Wilk test, *p* = 0.006). Therefore, the AD model was re-run with MEP log 10 as the dependent variable. In the final model in the AD group, there was still no significant relationship between CRQ and cortical excitability (R^2^_adj_ = 0.09, *p* = 0.286), and model residuals were normally distributed.

### 3.3. Effect of covariates

Table 2 shows the results of the exploratory nested regression of covariates with each group separately. No covariates significantly predicted MEP Amplitude when added to the model (*p*-values > 0.057). In the CU group, adding *age* to the model increased the beta-coefficient of CRQ by 31%, suggesting that accounting for *age* strengthens the effect of CRQ on MEP Amplitude. In the four AD participants with CSF, accounting for Aβ_42_, Phospho Tau, and Total Tau each CSF measure increased the beta-coefficient of CRQ by >100%, suggesting that at least in this small sample, accounting for amyloid and tau levels strengthens the relationship between CRQ and MEP Amplitude.

**TABLE 2:** Nested regressions.

	% Change in B_CRQ_	Change in p_CRQ_	Change in R^2^	Total R^2^ with covariate	*p*-value covariate
**(a) Cognitively unimpaired older adults.**					
Age	**30.59**	−0.015	0.17	0.53	0.057
Sex	10.62	−0.008	0.10	0.46	0.170
WTAR	3.96	−0.001	0.04	0.40	0.380
MMSE	3.85	−0.001	0.04	0.40	0.383
GDS	2.47	0.005	<0.01	0.36	0.794
Scalp-to-cortex distance	2.38	−0.005	0.11	0.47	0.137
LH cortical thickness	1.22	0.006	<0.01	0.36	0.877
ADCS-ADL	−16.06	0.015	0.21	0.60	0.098
Left precentral thickness	−16.57	0.019	0.13	0.49	0.100
**(b) Alzheimer’s disease.**					
pTau CSF (pg/mL)	**497.93**	−0.556	0.87	0.88	0.224
tTau CSF (pg/mL)	**476.20**	−0.424	0.72	0.74	0.343
Amyloid Tau Index (ATI) CSF	**440.08**	−0.697	0.96	0.97	0.109
A-beta 42 CSF (pg/mL)	**120.97**	−0.594	0.97	0.98	0.084
ADCS-ADL	12.85	−0.041	0.02	0.06	0.540
GDS	12.45	−0.026	0.01	0.03	0.740
WTAR	8.49	0.001	0.01	0.18	0.646
CDR-SB	6.57	−0.013	0.03	0.15	0.512
Sex	3.26	−0.006	0.01	0.05	0.628
MMSE	0.25	0.010	0.00	0.04	0.955
Left precentral thickness	−1.58	0.006	0.02	0.21	0.513
Scalp-to-cortex distance	−2.50	0.016	0.00	0.19	0.829
Age	−28.56	0.157	0.07	0.12	0.199

Within-group nested regression results are shown. Results are sorted by covariates with the greatest overall effect on the strength of the relationship between the cognitive reserve questionnaire and motor cortex excitability. A positive change in beta-coefficient of CRQ means that adding the covariate to the model increased the strength of the relationship between CRQ and MEP Amplitude. A negative change in beta-coefficient of CRQ means that adding the covariate to the model decreased the strength of the relationship between CRQ and MEP Amplitude. ATI, Amyloid Tau Index; computed as CSF A-beta 42/tTau; B_CRQ_, beta-coefficient of CRQ; p_CRQ_, *p*-value of CRQ; CSF, cerebrospinal fluid; GDS, geriatric depression scale; LH, left hemisphere; ADCS-ADL, Alzheimer’s Disease Cooperative Study – Activities of Daily Living scale; CDR-SB, clinical dementia rating scale sum of boxes; ATI, amyloid-tau index; WTAR, Weschler test of adult reading; MMSE, mini-mental state examination; SCD, scalp-to-cortex distance. Bold values indicate the change in B-covariate > 30%.

## 4. Discussion

This study investigated the relationship between CR and TMS markers of cortico-motor excitability in both CU and AD. Our primary model showed that the effect of CR on cortico-motor excitability was different between groups. In CU, higher CR was related to lower cortico-motor excitability. However, this relationship was not present in participants in the AD group (ranging in severity from MCI to mild dementia). Possible reasons for the lack of relationship in AD participants include abnormal cortical excitability in AD (Zadey et al., 2021), which may be particularly enhanced in the MCI stage (Dickerson et al., 2005). In line with this suggestion, the results of exploratory nested regression in the subset of AD participants with CSF values of Aβ_42_, Phospho Tau, and Total Tau strengthened the relationship between CR and cortical excitability. Future research is needed to determine how CR is related to cortical excitability in larger populations of older adults, and how this relationship is modified by the presence of amyloid and tau deposition in participants with preclinical AD.

The protective effects of CR has been established in multiple studies in normal aging, but show a more complex relationship in pathological aging. In cognitively normal older adults, higher cognitive reserve is related to better cognitive performance on tasks of memory, language, and executive function (Turcotte et al., 2022). Additionally, higher levels of education shows a neuroprotective effect, slowing accumulation of white matter hyperintensities and lacunes (Hotz et al., 2021). However, in AD, while higher cognitive reserve may delay the onset of clinically significant cognitive decline, higher CR is associated with faster disease progression after symptom onset (Soldan et al., 2017; van Loenhoud et al., 2019). In recent years, research has been aimed at uncovering the neurophysiologic underpinnings of the protective effects of cognitive reserve.

Our results show that, in CU, a higher score on the CRQ was related to lower cortico-motor excitability in M1. This is potentially consistent with the model of homeostatic disinhibition, which posits that adults with higher levels of CR may develop higher levels of both glutamatergic and GABAergic tone in their early adulthood (Gleichmann et al., 2011). GABA tone is an important regulator of excitatory/inhibitory balance and neuronal homeostasis, and therefore likely plays a significant role in modulating neural efficiency (Roth and Draguhn, 2012; Govindpani et al., 2017). MEP amplitudes are modulated by several neurotransmitters: they are reduced by GABA-A agonists such as benzodiazepines and increased by noradrenergic agonists (Ziemann, 2013). Therefore, a possible explanation of our results in CU is that decreased MEP amplitudes are driven by longstanding increases in GABA activity in CU participants with high levels of CR. Future work using paired-pulse TMS measurements may be able to experimentally address this question (Padovani et al., 2018).

Neurophysiologic correlates of CR in healthy older adults, including neural compensation and neural efficiency, have previously been investigated using fMRI and event-related potentials (ERPs). Task-based fMRI studies have implicated increased activity within the frontal lobes, temporal lobes, and default mode network as important brain regions supporting neural compensation and CR in older adults (Colangeli et al., 2016; Anthony and Lin, 2018). Unlike in younger participants, older adults show a positive correlation between greater BOLD patterns of activation related to CR and improved working memory, suggesting that the mechanisms subserving CR likely shift across the lifespan (Habeck et al., 2022). Additionally, ERPs in older adults with high CR also show evidence of increased neural efficiency, with less increase in ERP amplitude and latency with increasing task demand (Speer and Soldan, 2015; Gu et al., 2018). Our present findings that lower TMS-based measures of excitability are related to CR in CU participants are in line with the ERP literature. It is possible that TMS-based measures of excitability may serve as an additional measure of neural efficiency. Future studies using TMS-EEG should investigate cortical evoked responses in other heteromodal brain regions that are challenging to activate with fMRI or ERP experiments such as the parietal lobes, which may play an important role in the prodromal stages of AD (Ferreri et al., 2021, 2022; Perellón-Alfonso et al., 2022).

We found no relationship between CR and cortico-motor excitability in the AD group. The lack of a relationship was somewhat surprising given the known role that CR plays in clinical manifestations of AD, but is in line with similar results in the literature. In this context it is critical to remember that all our study participants showed signs of MCI or mild dementia, and may have sufficiently advanced disease pathology that their CR may have become insufficient to prevent clinical manifestations. Prior studies showing that greater ERP measures are related to higher CR in cognitively normal older adults have also found that there is no relationship between ERP measures and CR in patients with amnestic MCI (Speer and Soldan, 2015; Gu et al., 2018). One possibility is that the lack of association between CR proxies and cortical excitability measures in AD participants could reflect the breakdown of some of the protective neural mechanisms of CR. Task-based fMRI in AD largely shows increased brain activations in brain regions associated with higher CR, suggesting that neural compensation may be more important than neural efficiency in maintaining brain function once cognitive decline has begun (Anthony and Lin, 2018). Additionally, the protective effects of CR may vary in different stages of AD. For example, while high levels of CR can delay the onset of cognitive decline in patients with preclinical AD (Soldan et al., 2017), as the disease progresses, MCI patients with high levels of CR often show faster clinical decline (Soldan et al., 2017). If the protective effects of CR diminish as neuronal injury increases, there may be a threshold of neuronal injury after which time some mechanisms of CR may no longer function effectively (Soldan et al., 2020). Finally, our prior work has suggested abnormal cortico-motor excitability in AD (Zadey et al., 2021), there is a higher risk of seizures in AD (Beagle et al., 2017) which is linked to faster cognitive decline (Vossel et al., 2016). Future studies investigating the protective effects of CR in presymptomatic AD stages are needed to disentangle the relationship between CR, accumulation of proteins involved in neurodegeneration, and cortical excitability.

Our results should be interpreted in the context of several study limitations. Our sample size, particularly in the CU group (*n* = 15), was relatively small. Since the results in the CU group with R^2^_adj_ of 0.45 are supported by our power calculation, we feel confident that our results are supported by our sample size. Second, the age range of our participants is relatively wide, spanning younger to older seniors, and AD participants were slightly older than CUs. Future studies validating and extending our results with larger sample sizes separated by age range would be useful to confirm broader generalizability. Additionally, our cross-sectional design could have introduced biases in our analyses, and limited our ability to draw conclusions about longitudinal cognitive trajectories (Pfefferbaum and Sullivan, 2015). Future longitudinal studies are needed to see the extent to which CR affects changes in cortical excitability in healthy aging and AD. Next, while our cognitive reserve questionnaire has been validated in Spanish, it has not yet been externally validated in English. We have provided the full questionnaire in the Supplementary Annex so that additional validation can take place outside of our own lab. Our present dataset was underpowered to directly test for associations between CR and CSF amyloid and tau concentrations. Additional studies with longitudinal follow up, larger sample sizes, and the addition of Amyloid and Tau PET scans are needed to further investigate the mechanisms leading to alterations in cortical excitability and loss of the protective mechanisms of CR occurring in AD.

Strengths of this study include a use of TMS-EMG in a population of cognitively normal CU and AD participants. The CRQ assessed multiple domains of CR including education, occupation, social, and leisure factors. One limitation is that participants were drawn from several different studies, and that the CRQ was collected retrospectively in some participants. However, we do not expect this to affect the validity of our results since the CRQ is largely focused on childhood and occupational factors which are likely to be unchanged in older adulthood. Additionally, TMS stimulation protocols were similar enough across studies that comparable metrics of MEP amplitude could be analyzed jointly. Overall, the ability to pool data in this way confirms the robustness of our MEP and CRQ assessments across different protocols over time. Second, while AD participants were biomarker-confirmed, CSF and PET biomarkers were not available on CU participants, so it is possible that the CU cohort may have included some individuals with pre-symptomatic AD. However, we do not feel this affected the generalizability of our results in CU, as any presymptomatic AD participants in the CU group would only bias our results away from significance. Finally, CSF biomarker data was available on very few participants. Future studies with larger sample sizes and incorporation of Tau PET are needed to further understand the interactions between CR and the accumulation of proteins involved in AD-related neurodegeneration.

## 5. Conclusion

We found that lower cortical excitability is related to higher CR in the CU group, but not in the AD group. MEP amplitudes may reflect greater neural efficiency related to higher CR in cognitively normal older adults, perhaps related to alterations in glutamatergic and GABAergic tone. The reason for the lack of association seen in AD participants is unknown, but could reflect disruptions of the protective effects of CR. Future work is needed to better understand the neurophysiologic mechanisms leading to the protective effects of CR to elucidate, and eventually harness, the neural underpinnings supporting CR to promote healthy cognition in the aging brain.

## Data availability statement

The dataset from this study is property of Beth Israel Deaconess Medical Center (BIDMC) and contains sensitive medical information including a diagnosis of Alzheimer’s disease. In order to provide access to de-identified data from this dataset, BIDMC will require a data sharing agreement with a requesting investigator’s institution. Additionally, the BIDMC IRB would require acknowledgment that the receiver has obtained an exemption from their local ethics/IRB committee that any shared data is exempt from Human Subject Research. Therefore, the data can be accessed by reaching out to the Research Administrator of the BIDMC Neurology Department, who would then put the necessary agreements in place to facilitate data sharing.

## Ethics statement

The studies involving human participants were reviewed and approved by the Institutional Review Board of Beth Israel Deaconess Medical Center. The patients/participants provided their written informed consent to participate in this study.

## Author contributions

SB conceptualized the analysis, analyzed and interpreted the data, completed part of the Freesurfer image processing, and drafted and revised the manuscript. PF conceptualized the analysis, analyzed and interpreted the data, and revised the manuscript. JM and EZ acquired the data and revised the manuscript. VZ responsible for the additional Freesurfer image preprocessing and revised the manuscript. AP-L conceptualized the study, interpreted the data, and revised the manuscript. DB-F designed and conceptualized the study, oversaw the data collection, analyzed and interpreted the data, and made substantial contributions to revising the manuscript. ES conceptualized the study, interpreted the results, and revised the manuscript. All authors approved the final version of the manuscript for publication and agree to be accountable for the content of the work.

## Footnotes

1
http://surfer.nmr.mgh.harvard.edu/
