# Supplementary material for: Greater cognitive reserve is related to lower cortical excitability in healthy cognitive aging, but not in early clinical Alzheimer’s disease
Source: Front Hum Neurosci. 2023 Jul 27;17:1193407. doi: 10.3389/fnhum.2023.1193407 (PMC10413110; doi:10.3389/fnhum.2023.1193407)
Supplement: Supplementary file 1 [file Data_Sheet_1.PDF]

Subject Name:

Date:

BERENSON-ALLEN CENTER FOR NONINVASIVE BRAIN STIMULATION
BETH ISRAEL DEACONESS MEDICAL CENTER
330 Brookline Ave., Kirstein Building KS 158
TEL: 617 667-0203 • FAX: 617 975-5322

Dual Task Assessment

Instructions: *For each of the following sections, please circle the number corresponding to the answer that best represents you.*

Education and Culture

What is the highest level of education you completed?

0. Below high school
1. High school
2. College
3. Graduate

Did you have difficulty learning to read and write?

0. Yes, a lot of difficulty
1. Average amount of difficulty
2. No difficulty

Are you currently involved in an academic program or enrolled at a university?

0. No
1. Yes

If yes, please write the name and type of program: ____

While growing up, was there an educated environment where you were exposed to literature, art, or music?

0. No exposure
1. Some exposure
2. A lot of exposure

What is the highest level of education your father completed?

0. Below high school
1. High school
2. College
3. Graduate

What is the highest level of education your mother completed?

0. Below high school
1. High school
2. College
3. Graduate

Do you know a language other than English?

0. No
1. Some knowledge of another language
2. Very good knowledge of another language
3. Very good knowledge of 2 or more languages

Occupation

Please list your past jobs: ____

____

What did your past jobs involve? *(Circle all pertinent)*

0. No manual labor
1. Manual labor
2. Semi-skilled labor, secretary or technician (does not require higher education)
3. Professional, non-manager (requires higher education)
4. Professional, management

Leisure and Intellectual Activities

During your childhood did you read books?

0. No reading or some reading sporadically
1. Frequent reading (more than 3 hours per week)

Please estimate the total number of hours weekly that you spent reading as a child: ______________

Do you currently read?

0. No reading or some reading sporadically
1. Frequent reading (more than 3 hours per week)

Please estimate the total number of hours weekly that you currently spend reading: ______________

Did you ever learn to play a musical instrument?

0. No
1. Yes, I learned but no longer play
2. Yes, I learned to play and I still play it

If yes, which instrument? ____

If yes, did you study music in school or in a music academy or on your own? (*Please check one)*

☐In school or music academy ☐On my own

Are there any other activities that you consider “cognitive” or intellectual that you have participated in? (i.e. writing, painting, etc.)

0. No
1. Yes

If yes, please describe: ____

____

Physical Activities

During your childhood or adolescence were you involved in sports?

0. No
1. Yes

If yes, about how many hours a week did you play sports? ____________________________________

Do you currently practice a sport (including walking)?

0. No
1. Yes

If yes, about how many hours a week do you play sports? ____________________________________

Social Activities

During your life, do you think you have made a lot of friends?

0. Few friends
1. Average amount of friends
2. A lot of friends

How would you define how much you were involved in social events or activities during your life (i.e. going out with friends, participating in meetings/groups, volunteering)?

0. A Little
1. Average amount
2. A lot

Currently, how much are you involved in social activities?

0. A little
1. Average amount
2. A lot
